# The Presence of Serotonin in the Vestibular System: Supporting the Use of SSRIs/SNRIs in the Treatment of Vestibular Disorders—A Narrative Review

**DOI:** 10.3390/audiolres15060148

**Published:** 2025-11-06

**Authors:** Roberto Teggi, Daniela Caldirola, Giampiero Neri, Iacopo Cangiano, Pasquale Viola, Giuseppe Chiarella

**Affiliations:** 1 Department of Otolaryngology, IRCCS San Raffaele Scientific Institute, San Raffaele Hospital, Via Olgettina 60, 20132 Milan, Italy; 2 Department of Otolaryngology, Università Vita-Salute San Raffaele, 20123 Milan, Italy; 3 Department of Biomedical Sciences, Humanitas University, Via Rita Levi Montalcini 4, Pieve Emanuele, 20090 Milan, Italy; 4 Neurosciences Imaging and Clinical Sciences Department, University of Chieti-Pescara, 66100 Chieti, Italy; 5 Unit of Audiology, Phoniatrics and Vestibology, Regional Centre for Cochlear Implants and ENT Diseases, Department of Experimental and Clinical Medicine, University “Magna Graecia”, 88100 Catanzaro, Italy; pasqualeviola@unicz.it (P.V.);

**Keywords:** vertigo, serotonin, selective serotonin reuptake inhibitors (SSRI), selective norepinephrine reuptake inhibitors (SNRI), Menière’s disease (MD), vestibular migraine (VM), PPPD (persistent postural perceptual dizziness)

## Abstract

Background: Serotonin (5-HT) is a neurotransmitter and a hormone that regulates various functions. Serotonin receptors have been studied in animal experiments in the vestibular system, beginning from the inner ear and vestibular nuclei. However, the role of serotonin in the vestibular system and disorders remains to be clarified. Methods: A review of the literature was performed on different databases according to the PRISMA guidelines. Only publications published on humans and in English have been included. A total of 41 articles were included in this review. Results: There are many publications regarding the use of SSRI/SNRI in vestibular disorders. Regarding persistent postural perceptual dizziness (PPPD) and chronic subjective dizziness (CSD) the available evidence supports multimodality treatment incorporating vestibular rehabilitation, serotonergic medications, and cognitive behavior therapy, although most studies have not included a placebo control group. As for vestibular migraine (VM), SNRI and SSRIs were proposed as preventive therapy and demonstrated a reduction in vertigo attacks in patients with Menière’s Disease (MD), especially when symptoms of anxiety disorder were present. Conclusions: Although SSRIs/SNRIs are considered an off-label therapy for vertigo, several studies have assessed their efficacy in vestibular disorders, as indicated in the data published on PPPD, MD, and VM above all. As some studies report that serotonin receptors are also present in the inner ear and vestibular nuclei, it can be postulated that in cases where the natural levels of serotonin are altered, such as in depression and anxiety, the change in serotonin levels may affect vestibular function and play a role in vestibular disorders.

## 1. Introduction

Serotonin or 5-hydroxytryptamine (5-HT) is a neurotransmitter and a hormone that regulates various activities, including behavior, mood, memory, and gastrointestinal homeostasis [1]. In the last two decades, 15 serotonin receptors have been identified, which are grouped into seven families based on signaling mechanisms [2]. In the brain, serotonin exerts different functions, affecting mood, perception, reward, anger, aggression, appetite, memory, sexuality, and attention; in fact, it is difficult to find a human behavior that is not regulated by serotonin [3,4]. In the brain, different 5-HT receptors can also cause vasoconstriction or vasodilation in different vascular beds depending on the particular receptors that are expressed in each vessel wall [5]; for example, 5-HT1B receptors causes vasodilation, which may partly explain the analgesic effects of triptan antimigraine drugs [6]. For this reason, triptans are routinely used successfully in the treatment of migraine attacks. Recent studies seem to support their potential efficacy even in acute vestibular migraine attacks [7]. However, their use in vestibular migraine still requires more structured and extensive studies.

Outside the brain, 5-HT exerts different functions. For example, in the cardiovascular system, 5-HT has positive inotropic and chronotropic effects by increasing intracellular calcium in cardiac myocytes; it can be stored in platelet granules and plays a role in platelet aggregation [8].

At the ocular level, it can activate clear body muscles, thus provoking pupil dilation which in turn can lead to increased intraocular pressure. It can also act on the endocrine/metabolic system, increasing insulin secretion, glucose uptake in muscle tissue, lipogenesis in fat tissue, and lipid accumulation in the liver. Finally, in the gastrointestinal system, it can increase gastric emptying, gut motility, and intestinal secretion [9]. The action of serotonin on the brain and on other systems explains why an excessive activity of serotonin can lead to anxiety, agitation, confusion, tremor, hyperreflexia, and myoclonus, but also mydriasis, tachycardia, and tachypnea. These conditions are part of the so-called “serotonin syndrome”, which may occur when an SSRI is taken in combination with another medicine that also raises serotonin levels, such as another antidepressant or a triptane [10]. Serotonin receptors have been demonstrated in animal experiments in different parts of the vestibular system, beginning from the inner ear and vestibular ganglion. For example, in monkeys and rats, the expression of serotonin 5-HT1B and 5-HT1D receptors has been associated with vestibular ganglion cells in both the inferior and superior vestibular ganglia. In particular, 5-HT1B receptor immunoreactivity was associated with blood vessels in the vestibular ganglion. According to some authors, the parallel distribution of these serotonin receptors in the vestibular and trigeminal system may explain some mechanisms related to vestibular migraine [11]. Moreover, it has been demonstrated in a murine neurogenic migraine model that peripheral serotonin administration elicits extravasation of protein in the inner ear, concomitant with extravasation in meninges and other perivascular regions, thus underlying the possibility that central and peripheral serotonergic mechanisms in vestibular pathways may contribute to both balance and migraine disorders. In this regard, extravasation in the inner ear may explain one of the etiological links of vestibular migraine [12]. In animal experiments, caloric vestibular irrigation provoked increased serotonin release in the medial vestibular nucleus, unrelated to the irrigation of the auricle with ice water or hot water, thus underlying the possibility that 5-HT in the medial vestibular nucleus may be involved in the mechanism of vertigo, at least when induced by caloric stimulation [13]. Finally, the serotoninergic projections between the nucleus raphe and vestibular nuclei underline the possibility that raphe–vestibular connections are organized to selectively modulate processing in regions of the vestibular nuclear complex and may explain another possible pathophysiological mechanism of vestibular migraine [14]. Finally, some authors found a colocalization of 5-HT1F receptors and glutamate in the vestibular nuclei of rats, suggesting that the serotonin receptor may modulate glutamate release from the vestibular nuclei. Since the release of glutamate from trigeminal neurons has been implicated in migraines, it has been hypothesized that SSRIs may have a possible therapeutic use for vestibular migraine [15].

In addition, the serotonergic system plays a key modulatory role in several brain regions implicated in the regulation of fear, anxiety, and stress, such as the brainstem, amygdala, hippocampus, thalamus, insula, cerebellum, and prefrontal cortex [16]. By influencing neural activity across these areas, serotonergic signaling is involved not only in the regulation of anxiety, but also in vestibular processing, and this neurochemical overlap suggests a potential mechanism underlying the comorbidity between vestibular dysfunction and anxiety-related disorders [17]. Indeed, the vestibular system is tightly interconnected with cognitive–emotional circuits, and growing evidence from both animal models and human studies supports a bidirectional relationship between vestibular disturbances and emotional symptoms. Vestibular signals are integrated within networks that regulate emotional behavior, and disruptions in these pathways may contribute to the clinical presentation of anxiety in patients with vestibular disorders. The core of this circuitry is a parabrachial nucleus network, which presents a bidirectional relationship with the central amygdaloid nucleus, infralimbic cortex, and hypothalamus. Specifically, the parabrachial nucleus is a site of convergence of vestibular, somatic, and visceral information processing. Particularly, this pathway seems to be involved in avoidance, anxiety, and conditioned fear [18]. In line with this, anxiety disorders are considered as common precipitating conditions for persistent postural perceptual dizziness (PPPD) [19].

In recent years, selective serotonin reuptake inhibitors (SSRIs) have been the first line of medications used to treat moderate to severe anxiety disorders and depressive disorders; the mechanism of action involves blocking 5-HT, down-regulating presynaptic reuptake channels, thereby increasing synaptic concentrations of 5-HT. This class of drugs can regulate serotonin metabolism by acting on multiple targets. For example, one possible pharmacological target is the Serotonin Transporter (SERT). Its main function is to “reuptake” serotonin from the synaptic cleft, bringing it back into the presynaptic nerve ending after it has performed its action. This process is essential for regulating serotonin levels in the synapse. For example, Sertraline is a SSRI drug that works by blocking the SERT, inhibiting its function. This prevents serotonin reuptake, allowing it to remain in the synaptic cleft longer. This increase in serotonin concentration in the synaptic cleft enhances its action. SNRIs, on the other hand, act on both the SERT and the noradrenaline transporter, influencing the transmission of these two important neurotransmitters.

At first administration, SSRIs can have side effects, including headache, agitation, and mild irritability; they should be avoided in patients with bipolar disorders since they can trigger mania [20]. Examples of SSRIs widely used in clinical practice include the following: Fluoxetine, Paroxetine, Sertraline, Citalopram, Escitalopram, and Fluvoxamine.

Serotonin norepinephrine reuptake inhibitors (SNRIs) have been proposed more recently for the same psychiatric disorders. This category includes drugs such as: Duloxetine, Venlafaxine, Desvenlafaxine, and Milnacipran. Drugs like Venlafaxin and Duloxetine, in fact, also inhibit norepinephrine reuptake; they have been defined as more “stimulating”, although in some cases their therapeutic action may present more side effects at the beginning of therapy, above all paradoxical anxiety [20].

In fact, the emergence and development of SNRIs was driven by the possibility of a pharmacological class that, by acting on two neurotransmitters, achieved better results. On the other hand, the potential side effects were also greater for the same reason. Both pharmacological classes (SSRIs and SNRIs) were developed to outperform other drug categories, such as tricyclic antidepressants (TCAs), which had greater side effects.

Preliminary studies suggest that SSRIs and tricyclic antidepressants (TCAs) may be beneficial in improving postural control and vestibular symptoms in patients with anxiety symptoms or disorders, although more research is needed to establish the precise mechanisms and clinical efficacy of these treatments in this context [21].

In light of the information presented here, this narrative review aims to provide a preliminary overview of the use and potential usefulness of SSRIs and SNRIs in vestibular disorders, with the goal of identifying current opportunities, challenges, and gaps in the literature that warrant further investigation.

## 2. Materials and Methods

A comprehensive search of the literature was conducted in the main bibliographic databases (PubMed, SCOPUS) according to the “Preferred Reporting Items for Systematic Reviews and Meta-analyses” (PRISMA) guidelines. We used the following strings: “Vestibular disorders and SSRIs”, “Vestibular Disorders and Selective Serotonin Reuptake Inhibitors”, “SSRIs and vestibular migraine” “Selective Serotonin Reuptake Inhibitor and vestibular migraine” “SSRIs and Perceptive Postural Perceptual Vertigo”, “SSRIs and Chronic Dizziness”, “SSRIs and Vertigo”. A total of 113 articles were found.

The inclusion criteria were the following: peer-reviewed articles on humans, with no age restrictions, written in English, including clinical trials and reviews. The search was limited to articles published in the last 25 years (2000–2025) to ensure that the latest research was included.

The exclusion criteria were as follows: non-peer-reviewed articles, studies that were case reports, editorials, and letters to the Editor without comprehensive results.

All authors discussed the search results at each stage and reached consensus on the relevance and inclusion of the studies identified.

**Figure 1:**
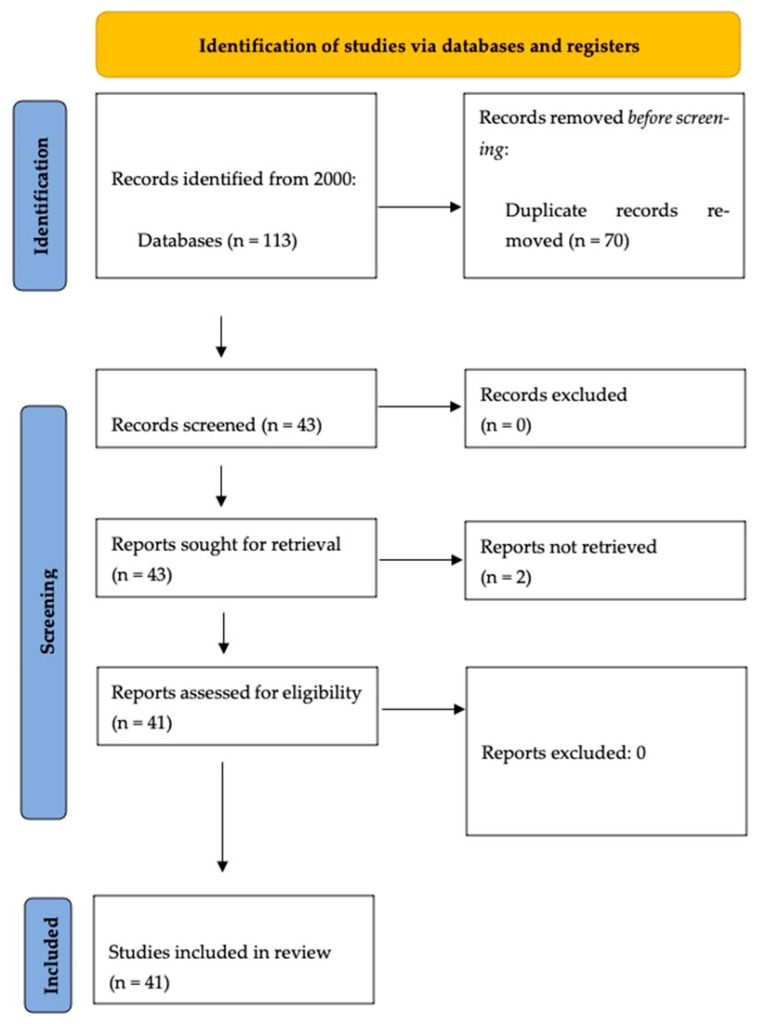
Search strategy flowchart; another study has been included, published before 2000, explaining pathophysiological bases of overlap between anxiety and vertigo.

From an initial pool of 113 studies, 41 studies were included in this review, as summarized in Figure 1.

## 3. Results

The following sections provide a general overview of the main findings from the reviewed studies, organized by key areas of interest.

### 3.1. SSRIs in Persistent Postural Perceptual Dizziness and Chronic Subjective Dizziness

Diagnostic criteria for PPPD have been defined by the Barany Society in 2017; it is a chronic disorder characterized by dizziness, unsteadiness, or non-spinning vertigo that lasted for 3 months or more, with symptoms exacerbated by upright posture, active or passive motion of self, and exposure to environments with complex or moving visual stimuli [22]. It has been defined as a maladaptive condition to an initial disorder provoking changes in the balance system.

Ongoing research supports multimodal treatment plans incorporating vestibular rehabilitation, serotonergic medications, and cognitive behavior therapy [23]. While many studies can be found on defined clinical conditions preceding PPPD and those having clinical overlap with it, like chronic subjective dizziness (CSD), few placebo-controlled studies have been published on PPPD. Moreover, studies have often carried out multiple interventions (i.e., SSRIs and rehabilitation or cognitive behavioral therapy) but without a placebo group [24].

Recently, Bo Tang et al. compared two groups treated with SSRIs, the first without organized exercise, and the second received public dancing lessons as a form of rehabilitation. Evaluation was carried out through questionnaires including the Dizziness Handicap Inventory (DHI), Hospital Anxiety and Depression Scale (HADS), Active-specific Balance Confidence Scale (ABC), and Vestibular Disorder Activities of Daily Living Scale (VADL). Both groups improved at 3 and 6 months, although those also receiving public dance training had better results [25].

Two further works reported that SSRIs and SNRIs are among the most prescribed therapeutic measure for PPPD. They usually need titrating the dose up from a low starting level to reach the therapeutic range. Both classes of drug have a slow onset of action and it may take several weeks before any benefit is seen. Possible modes of action may include improving psychological symptoms, such as anxiety, that are present in many people with PPPD, or they may have direct effects on the widespread balance network in the brain [26,27].

Other authors proposed, among other interventions, SSRIs in a group of PPPD subjects of pediatric age (mean age 14.6 years); they found that the group of subjects undergoing drug therapy in association with cognitive behavioral therapy had better results. They also reported that SSRIs were well tolerated in these patients [27].

As previously stated, different investigations have assessed the utility of SSRIs as a form of therapy for CSD.

Staab et al. treated 60 subjects divided into three groups for at least 20 weeks with SSRIs: idiopathic dizziness, psychogenic dizziness, and dizziness related to a neurological disorder with a psychological overlap. They had previously been treated with other antivertiginous drugs with no effect. In the total sample, 63% of subjects significantly improved; in particular, patients whose only diagnosis was a psychiatric disorder and those with coexisting peripheral vestibular conditions or migraine headaches had better outcomes than patients with central nervous system deficits [28].

In 2004, the same author published new data on 24 subjects with a diagnosis of chronic dizziness; 18 also presented a diagnosis of major anxiety. Patients were treated with Sertraline, beginning from 25 mg a day and increasing until 200 mg as needed (median dose was 100 mg). Dizziness, functional impairment, and psychological distress were measured using the DHI and Brief Symptom Inventory-53 (BSI-53). It was found that 73% of patients had significant benefit on both dizziness and anxiety, while six subjects reported a full remission of symptoms [28].

Horii et al. administered 20 mg Paroxetine to a group of 47 subjects with chronic dizziness and a comorbidity for anxiety. Their evaluation was performed with different questionnaires, which yielded good results in reducing anxiety and other psychiatric disorder but with lower efficacy on dizziness [29].

The same authors also reported that the use of these drugs in patients with anxiety/depression and self-reported dizziness showed improvement in their symptoms in a similar way. Within this study, patients were administered Milnacipran and compared to the results of a previous study that had patients treated with Fluvoxamine. The rate of patients with a post/pre ratio of handicaps < 80% was higher in the Milnaciplan group compared with the Fluvoxamine group [30].

### 3.2. SSRIs in Other Vestibular Disorders

SSRIs and SNRIs have been proposed as preventive therapy for migraines in the last 20 years, although their efficacy is still under debate [31]. Two recent studies have been published in which Venlafaxine was proposed as a preventive therapy for vestibular migraine (VM).

Salviz et al. carried out a randomized clinical trial comparing the effectiveness of Venlafaxine to Propranolol by evaluating the number of vertigo spells and a series of questionnaires for both dizziness and anxiety; both groups significantly decreased the number of vertigo spells and no difference was detected between groups, although patients taking Venlafaxine demonstrated a lower level of anxiety and depression as assessed with the Beck Anxiety and Depression Inventory [32].

Similar results were also found by Liu et al. who evaluated the efficacy and safety of Venlafaxine, Flunarizine, and Valproic Acid in a randomized comparative trial for VM prophylaxis. They reported that all therapies were well tolerated and significantly decreased the number of vertigo attacks and Venlafaxine and Valproic Acid showed better efficacy than Flunarizine. Venlafaxine demonstrated an advantage in terms of emotional domains [33].

SSRIs have also been shown to be useful in reducing vertigo attacks in patients with Menière’s disease (MD), especially when symptoms of generalized anxiety disorder were present [34].

Kıroğlu et al. treated a group of 12 subjects with MD and anxiety with Escitalopram 10 mg, who had been previously treated with different drugs without results; in all subjects, no vertigo attack was observed during the observation period. In contrast, no efficacy was found on hearing levels [34]. Benefits of SSRIs in MD have also been reported in another study in which three patients with an anxiety comorbidity, previously treated with other drugs, were administered Sertraline 50 mg/day; all patients reported complete control of vertigo spells [35].

Finally, SSRIs have been used in tandem with Clonazepam and a rehabilitation program for a patient with mal de debarquement syndrome, demonstrating complete resolution of symptoms [36].

## 4. Discussion

Although SSRIs/SNRIs are an off-label therapy for vertigo, several studies have assessed the efficacy of these drugs in treating vertigo. The drugs are normally well tolerated, even in pediatric [27] and elderly patients [37].

Since PPPD is a maladaptive condition, in which anxiety more probably plays an important role, the efficacy of SSRIs/SNRIs may be explained by the pathophysiology of the disorder [19].

Many studies involving SSRIs and SNRIs in individuals with vestibular or balance disorders have focused on conditions where the connection between the vestibular system and cognitive–emotional circuits appears to play a significant role in the clinical presentation [18,38,39], such as PPPD. Given that PPPD is a maladaptive condition in which anxiety and emotional dysregulation likely contribute to symptoms, it is plausible that the effectiveness of SSRIs and SNRIs is related to their modulatory effects on the integrated vestibular–emotional neural networks involved in the pathophysiology of the disorder.

Regarding the effectiveness of these drugs in vestibular disorders (MD and VM above all), in our opinion, two possibilities should be considered. Since patients with vertigo more easily develop anxiety, which in turn may facilitate vertigo attacks, reducing psychiatric comorbidity may lead to a reduced facilitation to a vertigo attack [40,41].

On the other hand, since other studies have reported that serotonin receptors are also present in the inner ear and in the vestibular nuclei [3,11,12], a direct relationship can be postulated between increased serotonin levels and vestibular disorders. In a recent investigation, serum levels of chromogranin-A (Cg-A), which is stored and co-released with serotonin, was measured in a group of patients with MD. A correlation has been found between the number of vertigo spells and Cg-A levels; however, according to the authors, the finding is not inconsistent with the hypothesis of an involvement of serotonin in the pathophysiology of vertigo [41].

Finally, during an abrupt discontinuation of SSRIs, some patients report that dizziness is among the most annoying symptoms. The pathophysiology of SSRI discontinuation syndrome is still under debate. Abrupt discontinuation of an SSRI is likely to result in a decrease in serotonin levels in the ventral nerve cord, as in many other areas of the brain [42]; since serotonin receptors are present in the vestibular nuclei, it may also been hypothesized that changes in serotonin levels in vestibular nuclei may be the cause of dizziness following SSRI withdrawal [43]. If demonstrated, this suggests that in cases where the natural levels of serotonin in the brain are altered, such as in depression and anxiety, the change in serotonin levels in the ventral nerve cord may affect vestibular function.

## 5. Conclusions

Recent publications support the hypothesis that SSRIs can be useful in the treatment of vestibular disorders, and not only PPPD. The reciprocal connections between the vestibular system and neural circuits involved in anxiety may explain this possibility. All studies underline that these drugs are generally well tolerated, even in pediatric and elderly patients. Normally, the minimum therapeutic dose is to be preferred. Moreover, they can be administered in association with other specific therapies for vertigo, and in some patients, better results are likely obtained when used in conjunction with an active lifestyle and/or physiotherapy. It should be noted, on the other hand, that most studies report results on small sample sizes in which multiple interventions are performed. To assess therapeutic efficacy of these drugs in vertigo treatment, more specific studies on larger sample sizes are required.
